# Anti-pA137R antibodies exacerbate the pathogenicity of African swine fever virus in pigs

**DOI:** 10.1128/jvi.00172-25

**Published:** 2025-05-29

**Authors:** Huanjie Zhai, Yuxuan Gao, Yuanmao Zhu, Qinghe Hou, Nian Wan, Tao Wang, Su Li, Dongming Zhao, Hua-Ji Qiu, Yongfeng Li

**Affiliations:** 1 State Key Laboratory for Animal Disease Control and Prevention, National High Containment Facilities for Animal Diseases Control and Prevention, Harbin Veterinary Research Institute, CAAS 687216 , Harbin, China; Lerner Research Institute, Cleveland Clinic, Cleveland, Ohio, USA

**Keywords:** African swine fever virus, antibody-dependent enhancement, anti-pA137R antibodies, Fc*γ *receptors, pathogenicity

## Abstract

African swine fever virus (ASFV) is the causative agent of African swine fever (ASF), a highly contagious disease with a mortality of up to 100% in pigs. Currently, no safe and effective vaccines are available globally, except for two licensed vaccines in Vietnam. One of the factors impeding the development of vaccines is antibody-dependent enhancement (ADE). Our previous study showed that the antibodies against the ASFV protein A137R (pA137R) could promote ADE of ASFV infection in primary porcine alveolar macrophages. However, direct evidence of ADE in target animals remains limited. Here, we investigated whether anti-pA137R antibodies exacerbate ASFV pathogenicity in pigs. The immunization-challenge experiment showed that the pigs immunized with pA137R exhibited more severe clinical signs and died earlier than the unimmunized ones. Meanwhile, the examination of viral replication in the blood and various tissues revealed that the anti-pA137R antibodies promoted the infectivity of ASFV. Notably, the production of interferon alpha was significantly upregulated in the blood of the immunized piglets, while the level of interferon beta, tumor necrosis factor alpha, and interleukin 1beta remained unchanged. Mechanistically, the Fc gamma receptor (Fc*γ*R) II and Fc*γ*RIII facilitated ADE of ASFV infection in the PK-15 cells overexpressing the receptors. Taken together, our study demonstrates for the first time that the anti-pA137R antibodies enhance ASFV pathogenicity in pigs, providing novel insights into the role of anti-ASFV antibodies in the pathogenesis of ASFV and the rational design of innovative ASF vaccines.

**IMPORTANCE:**

The antibody-dependent enhancement (ADE) effect can augment viral replication or elicit aberrant immune responses, ultimately aggravating the disease progression. Recently, we have shown that the antibodies against A137R protein (pA137R) of African swine fever virus (ASFV) can drive ADE *in vitro*. The present study shows that the anti-pA137R antibodies can enhance viral infection and exacerbate clinical signs in pigs. Importantly, the aberration in interferon alpha production might be related to the pathogenicity of ASFV mediated by ADE. Mechanistically, Fc gamma receptor (Fc*γ*R) II and Fc*γ*RIII are shown to facilitate ASFV infection. This study is the first to demonstrate that the anti-pA137R antibodies enhance the pathogenicity of ASFV in pigs, offering novel insights into the pathophysiology of ASFV and the development of African swine fever vaccines.

## INTRODUCTION

African swine fever (ASF), a highly contagious disease, poses an unprecedented and catastrophic threat to the global pig industry. Despite the conditional use of two licensed ASF vaccines in Vietnam, there remains an urgent need for a universally safe and effective vaccine against ASF. Unfortunately, some inactivated ASF vaccines and subunit vaccines may exacerbate disease severity and significantly increase mortality in pigs following virulent ASFV challenge (1–3). African swine fever virus (ASFV), the causative agent of ASF, targets monocytes and macrophages. Like severe acute respiratory syndrome coronavirus 2 (SARS-CoV-2) and porcine reproductive and respiratory syndrome virus (PRRSV) (4, 5), ASFV fails to induce traditional neutralizing antibodies (NAbs), implying its potential to drive antibody-dependent enhancement (ADE), which may represent a pivotal challenge impeding ASF vaccine development.

The ADE effect refers to a phenomenon in which re-infection of pre-exposed animals with the same virus species enhances viral replication or elicits abnormal immune responses, ultimately aggravating disease progression. It has been shown that infections with dengue virus (DENV) or Zika virus (ZIKV) can elicit cross-reactive antibody responses, and virtually all anti-DENV antibodies contribute to ZIKV ADE (6). Notably, a study has revealed that the anti-DENV antibodies potentiate ZIKV replication in pregnant mice, resulting in increased placental damage, fetal growth retardation, and fetal reabsorption (7). Furthermore, it has been documented that patients’ anti-human immunodeficiency virus (HIV) lgA can bind to HIV particles and adhere to the surface of monocytes and mucosal macrophages, thereby promoting virus invasion and enhancing virus replication *in vivo* (8). However, the evidence regarding the ADE of ASFV infection in pigs is still scarce.

pA137R, previously known as p11.5, is a crucial constituent of icosahedral particles of ASFV and plays multiple roles in viral replication and pathogenesis. In viral factories, it colocalizes and interacts with p72, thereby facilitating ASFV replication (9). Moreover, pA137R assembles into a dodecahedron cage structure and is responsible for the production of infectious ASFV particles (10). Specifically, anti-pA137R monoclonal antibodies exhibit remarkable efficacy in capturing and targeting ASFV (11). Beyond its structural significance, the *A137R* gene encodes a virulence-associated protein that exerts its effect by inhibiting the interferon signaling cascade, orchestrated by the cyclic GMP-AMP synthase-stimulator of interferon genes pathway (12, 13). Several studies have demonstrated that the underlying mechanism of ADE is that virus-specific antibodies can augment the entry of viruses into host cells, thereby facilitating the production of infectious viral particles (14–16). Anti-pA137R antibodies have been shown to enhance the *in vitro* replication of ASFV (17, 18). Consequently, it is imperative to thoroughly evaluate whether the antibodies against pA137R enhance the pathogenicity of ASFV infection in pigs.

The Fc gamma receptors (Fc*γ*Rs) on the cell surface are pivotal for the FcRs-mediated ADE. Human cells express three distinct types of Fc*γ*Rs, i.e., Fc*γ*RI (CD64), Fc*γ*RII (CD32), and Fc*γ*RIII (CD16). With the involvement of Fc*γ*Rs, particularly Fc*γ*RIIa and Fc*γ*RIIIa, the virus adsorption and internalization are potentiated with treatment of non-NAbs or sub-NAbs, resulting in an augmenting infection (19). Particularly, Fc*γ*RII and Fc*γ*RIII have been implicated in the ADE phenomenon observed in SARS-CoV-2 infection (4), while Fc*γ*RII mediates ADE of PRRSV infection (20). Our previous study has reported that the anti-pA137R antibodies interact with ASFV to form a virus-antibody complex, binding to primary porcine alveolar macrophages (PAMs), through Fc*γ*RII and Fc*γ*RIII to mediate the ADE of ASFV infection on PAMs (17).

This study aimed to investigate whether anti-pA137R antibodies enhance ASFV pathogenicity in pigs. Here, we demonstrate the first time that the antibodies targeting the structural protein pA137R aggravate ASFV pathogenicity in pigs.

## RESULTS

### Immunization with pA137R induces **antibody production** in pigs

**Fig 1:**
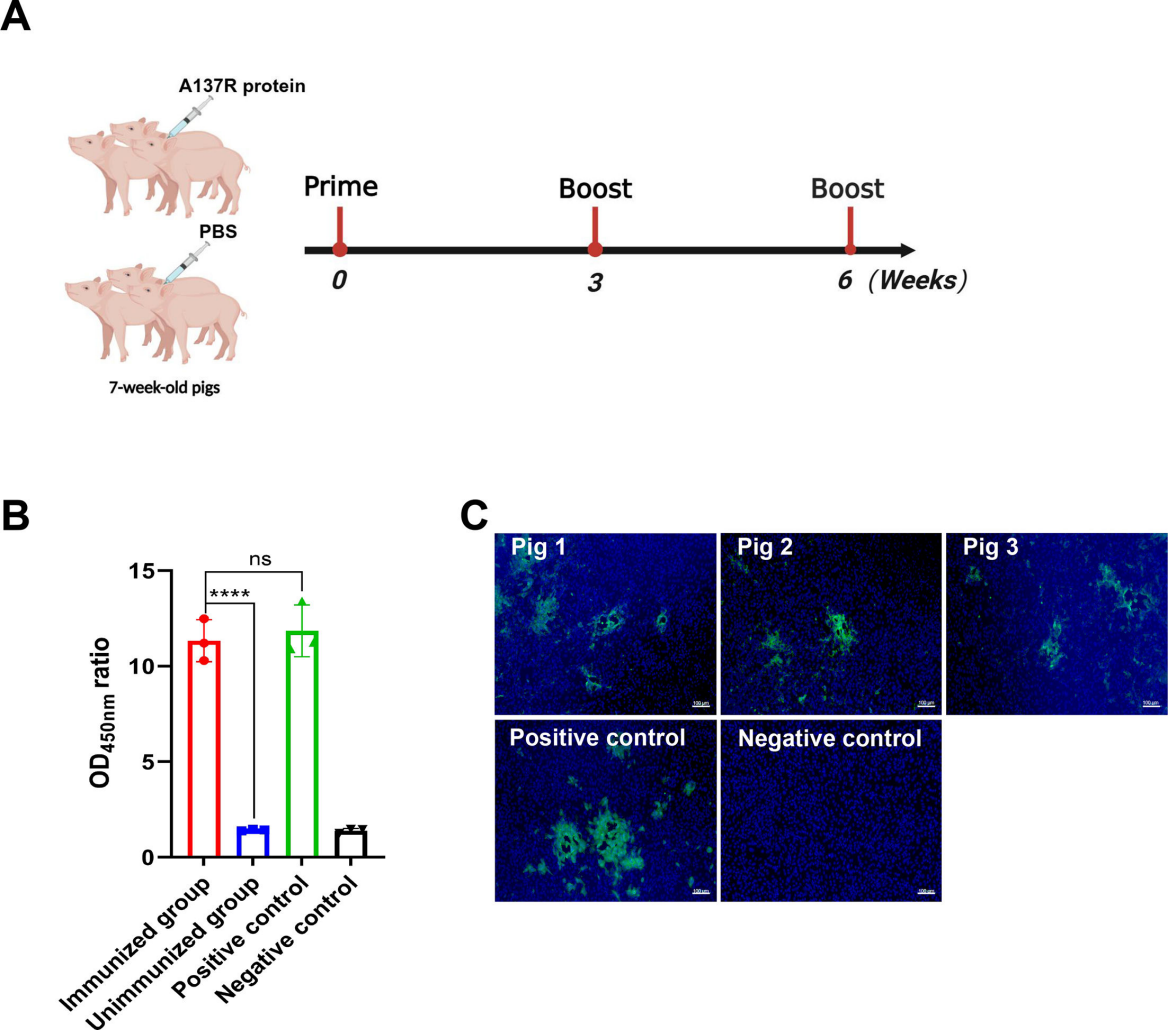
Anti-pA137R antibodies are detectable in the sera from the pA137R-immunized pigs. (**A**) Schematic diagram of immunization strategy. The pigs were immunized with pA137R, followed with two booster vaccinations at 3-week intervals. Sera were collected periodically. Anti-pA137R antibodies are induced in pA137R-immunized pigs. Anti-pA137R antibodies were tested by ELISA (**B**) and IFA (**C**).

After the pigs were immunized three times with pA137R at 0, 3, and 6 weeks (Fig. 1A), the serum samples of the immunized pigs were collected and assessed by indirect enzyme-linked immunosorbent assay (ELISA), with ASFV-convalescent pig sera serving as a positive control and specific pathogen-free (SPF) pig sera as a negative control. The results indicated that specific antibodies against ASFV pA137R were present in the pA137R-immunized pig sera (Fig. 1B). Following infection of wild boar lung (WSL) cells with ASFV-HLJ/18 strain, the sera obtained from the pigs immunized with pA137R were introduced into the cells. Subsequently, immunofluorescence assay (IFA) detection revealed that pA137R was recognized by the pig sera (Fig. 1C).

### Anti-pA137R antibodies exacerbate the clinical signs **of the ASFV-infected pigs**

It has been found that certain serum components of natural ASFV infection can increase the infectivity of ASFV (2). Our recent study has also shown that the anti-pA137R antibodies contribute to the enhancement of ASFV replication *in vitro* (17). To evaluate the potential role of anti-pA137R antibodies in the pathogenesis of ASFV, the piglets were divided into two groups: an immunized group vaccinated with pA137R and an unimmunized group vaccinated with the phosphate-buffered saline (PBS). Both groups were subsequently challenged with 10^3.0^ 50% hemadsorbing doses (HAD_50_) of the ASFV HLJ/18 strain. Daily body temperature and clinical signs were monitored.

**Fig 2:**
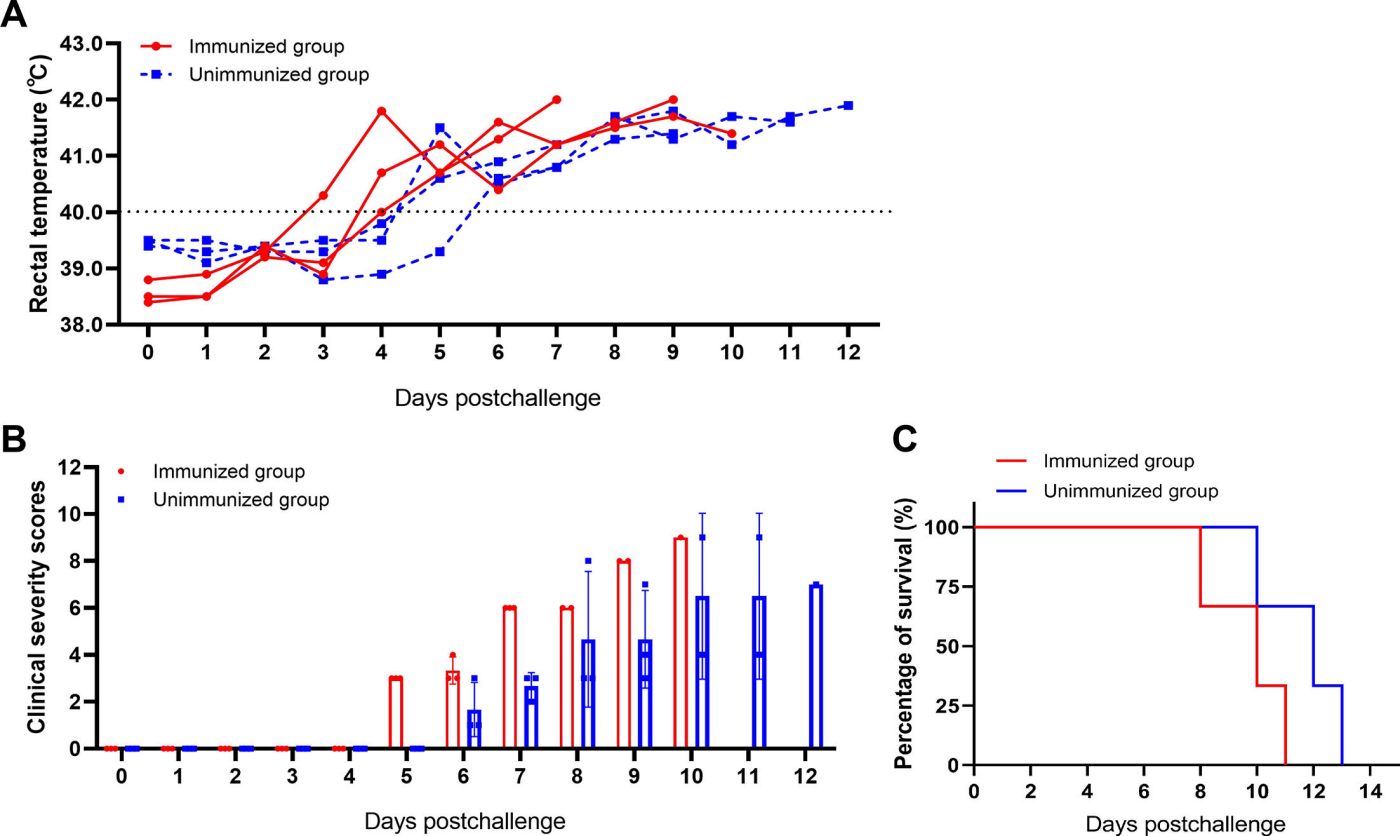
Anti-pA137R antibodies aggravate the clinical signs and death of the ASFV-infected pigs. The pA137R-immunized pigs were infected with 10^3.0^ HAD_50_ of the ASFV HLJ/18 strain and monitored daily until death. The rectal temperatures (**A**), clinical scores (**B**), and survival rates (**C**) of all the pigs were assessed daily after the challenge. The dashed black lines in panels A indicate the threshold of normal rectal temperature (40°C). The scores were calculated as described previously (21, 22).

Interestingly, one immunized piglet exhibited a fever (>40°C) as early as 3 days postchallenge (dpc), exceeding 40°C, and all the immunized piglets experiencing elevated body temperature at 4 dpc. In contrast, the unimmunized group displayed a delayed onset of hyperthermia, with fever at 5 dpc (Fig. 2A).

Following the onset of hyperthermia, all piglets exhibited a series of clinical manifestations typical of ASF, including anorexia, lethargy, and cutaneous hemorrhages. The clinical signs associated with ASF progressively worsened as the viral infection progressed. Subsequent clinical score evaluations showed that the immunized pigs exhibited ASF-specific clinical signs earlier and had higher clinical scores (Fig. 2B), suggesting more severe disease progression compared with the unimmunized pigs (21).

Several studies have shown that vaccination with some ASF vaccine candidates accelerates the mortality of pigs (1–3). The immunized piglets succumbed to the infection between 8 and 11 dpc, whereas the unimmunized piglets died between 10 and 13 dpc (Fig. 2C). These findings suggest that the anti-pA137R antibodies may shorten the time to death of the ASFV-infected piglets, emphasizing the role of anti-pA137R antibodies in enhancing the pathogenicity of ASFV in pigs.

### ASFV replication is significantly enhanced in the pA137R-immunized pigs

**Fig 3:**
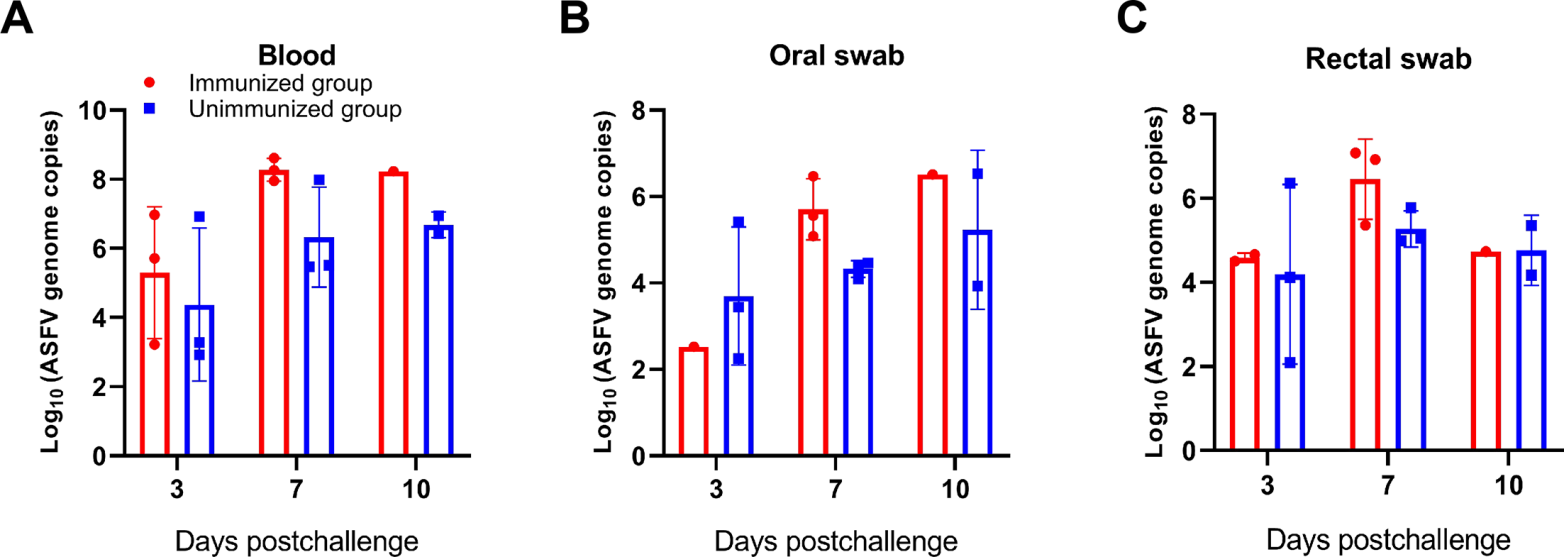
Anti-pA137R antibodies enhance ASFV replication in pigs. Blood samples (**A**), oral swabs (**B**), and rectal swabs (**C**) were collected at the indicated times postinfection and subjected to qPCR to quantify the viral genome copies.

To evaluate the replicative capacity of ASFV in pigs, we collected blood samples at 3, 7, and 10 dpc and quantified the viral genome copies by quantitative real-time PCR (qPCR). As shown in Fig. 3A, the immunized pigs consistently exhibited higher ASFV replication levels at indicated times compared with the unimmunized animals. These results suggest that the anti-pA137R antibodies augment ASFV replication *in vivo*, highlighting the complex interplay between antibodies and ASFV.

To further elucidate the viral transmission potential among pigs, we subsequently collected oral and anal swabs at 3, 7, and 10 dpc and analyzed the viral loads. Notably, the viral genome copies were quantified in oral (Fig. 3B) and rectal (Fig. 3C) swabs from both the immunized and unimmunized groups at the earliest time point of 3 dpc, with the viral load progressively increasing throughout infection.

### Anti-pA137R antibodies are associated with severe histopathological lesions in the ASFV-challenged **pigs**

**Fig 4:**
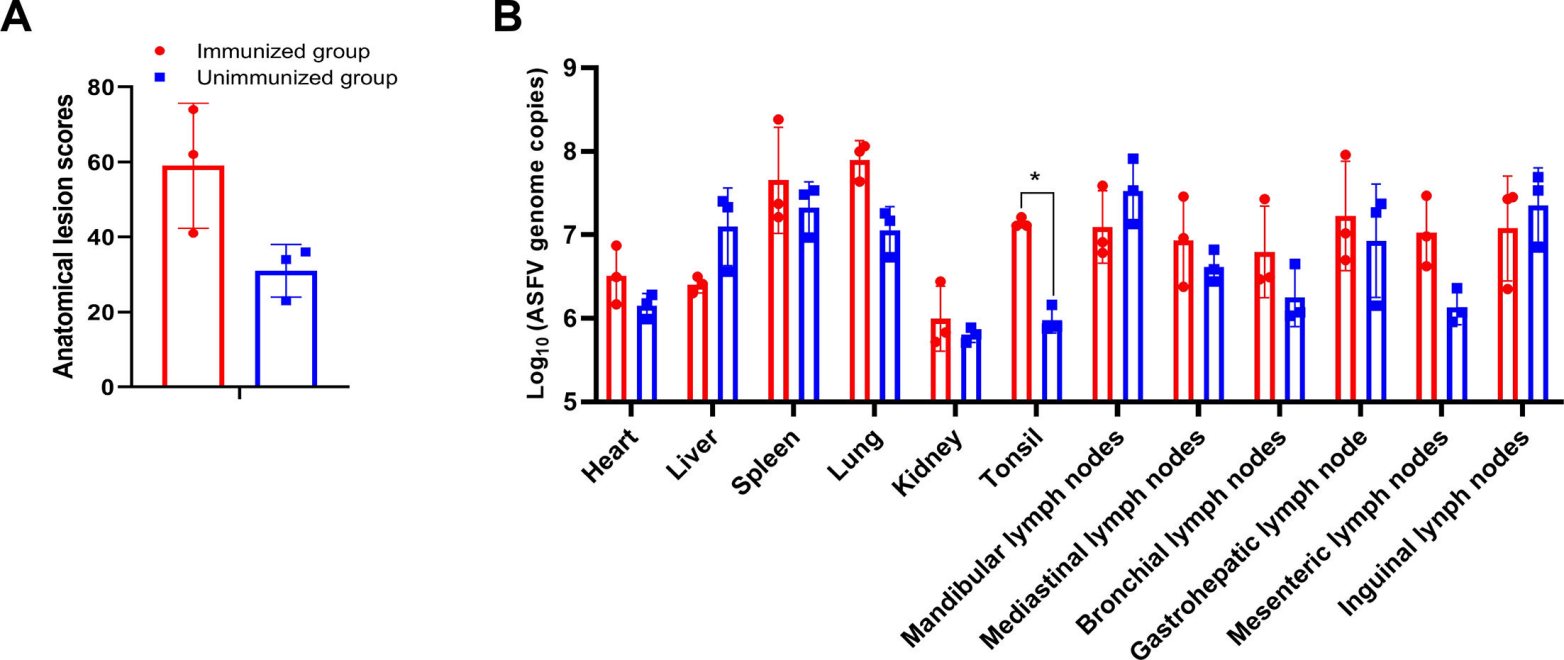
Anti-pA137R antibodies aggravate the histopathological lesions of the pigs infected with ASFV. (**A**) Increasing histopathological lesions. The anatomical lesion scores were recorded and summed daily based on a harmonized scoring system described previously (22) (**B**) Enhancement of ASFV replication in various tissues of the ASFV-infected pigs. Tissue samples from the infected pigs at necropsy were systematically procured, including the heart, liver, spleen, lung, kidney, tonsils, and an extensive array of six lymph nodes. All the tissues were collected from all the infected pigs and subjected to qPCR analysis to quantify the genome copies.

Highly virulent ASFV infection can cause extensive tissue damage and lesions in pigs (23). In the present study, all piglets infected with the ASFV HLJ/18 strain exhibited typical ASF clinical signs, such as severe congestion and multifocal diffuse hemorrhages in all tissue samples. The lesions included hemorrhagic spots on the heart, an enlarged and bruised liver, and hemorrhages within the gallbladder wall, lungs, kidneys, inguinal lymph nodes, and intestinal lymph nodes. Additionally, the spleen displayed blackness, swelling, fragility, and mesenchymal hyperplasia. It is noteworthy that the immunized pigs had higher scores in tissue lesion assessment (Fig. 4A), suggesting that the anti-pA137R antibodies aggravate ASF pathological lesions.

To investigate ASFV replication in the tissues, a comprehensive panel of tissues, including the heart, liver, spleen, lungs, tonsils, and various lymph nodes, was collected from all the deceased piglets. Subsequently, qPCR was utilized to accurately quantify the viral genome copies in these tissues. As depicted in Fig. 4B, both immunized and unimmunized piglets exhibited viral genome copies exceeding 10^6.0^/mL, indicating substantial viral presence. Especially, the tonsils of the immunized piglets harbored a significantly higher viral load compared with the unimmunized group. These findings imply that the administration of anti-pA137R antibodies to piglets may enhance ASFV replication in various tissues.

### **The ADE induced by anti-pA137R antibodies increases interferon alpha production in ASFV-infect**ed pigs

**Fig 5:**
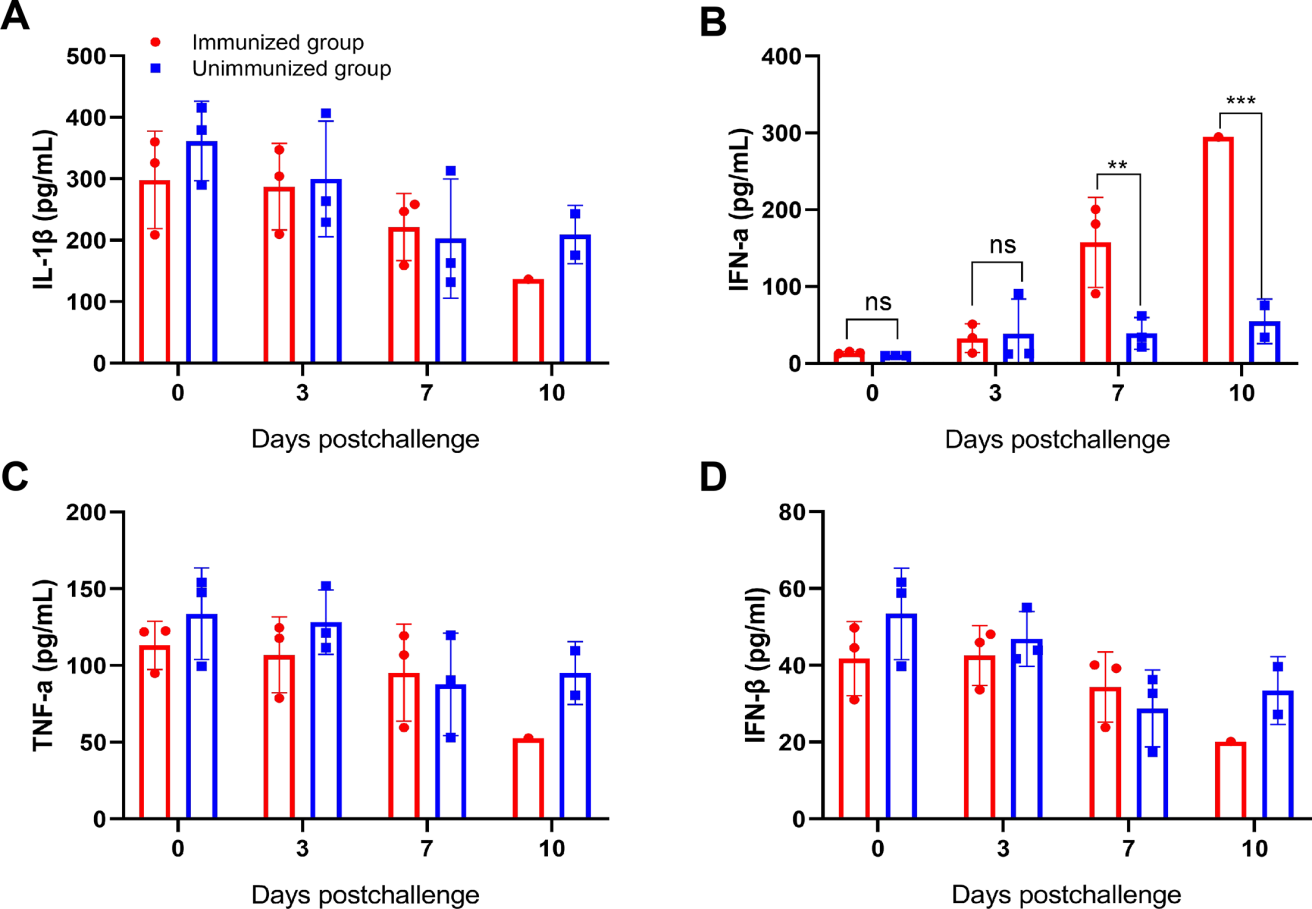
ADE of ASFV infection leads to increased IFN-*α* production in the pA137R-immunized pigs. The plasma samples were collected at indicated times and analyzed for the expression of cytokines, including IL-1*β* (**A**), IFN-*α* (**B**), TNF-*α* (**C**), and IFN-*β* (**D**). Statistical analysis was performed using one-way ANOVA followed by Student’s *t* test: ****, *P* < 0.0001; ***, *P* < 0.001; **, *P* < 0.01; *, *P* < 0.05. The data were presented as means and SDs.

ASFV infection usually induces an inflammatory storm in immune cells, characterized by abnormal production of cytokines in immune cells, including interleukin 1beta (IL-1*β*), IL-6, IL-12, IL-18, tumor necrosis factor alpha (TNF-*α*), and C-C motif chemokine ligand 4 (CCL4) (24–26). However, it remains unclear whether the ADE of ASFV infection contributes to the production of inflammatory cytokines in pigs. The blood samples from the immunized and control pigs were collected at the indicated times postchallenge and subsequently analyzed for cytokine and chemokine profiles. The results showed that the production of interferon alpha (IFN-*α*) was at a higher level in the blood of the immunized-challenged pigs compared with the control pigs, whereas the production of IL-1*β*, TNF-*α,* and IFN-*β* remained unchanged (Fig. 5). These results indicate that the ADE driven by anti-pA137R antibodies leads to increased IFN-*α* production in pigs, suggesting that high levels of IFN-*α* contributes to the pathogenicity of ASFV in pigs.

### Fc*γ*RII and Fc*γ*RIII are involved in promoting ASFV infection upon the treatment with anti-pA137R antibodies

**Fig 6:**
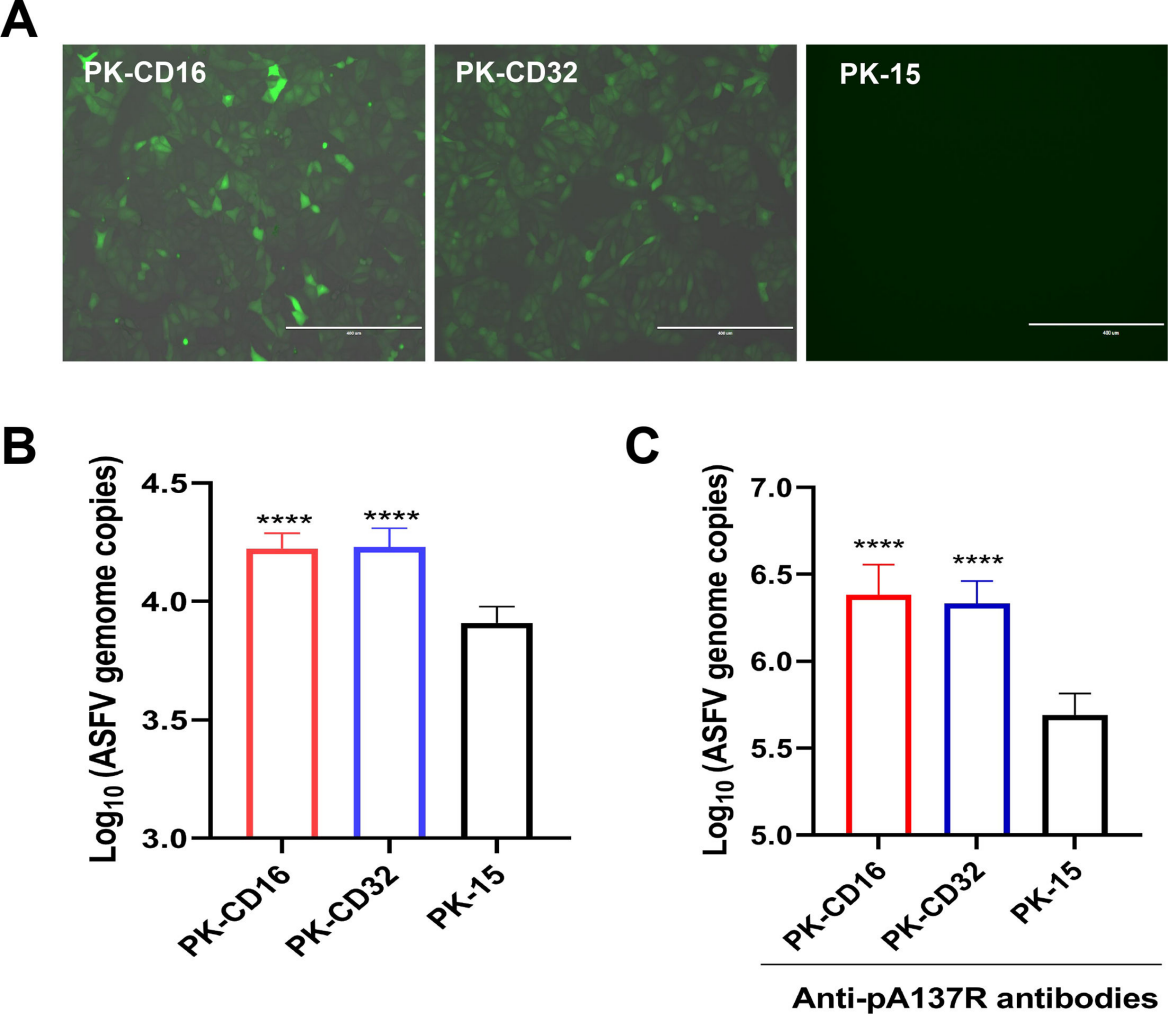
Anti-pA137R antibodies promote ASFV replication via Fc*γ*RII and Fc*γ*RIII. (**A**) Establishment of a PK-15 cell lines stably expressing CD16 or CD32. (**B**) Enhanced ASFV replication in PK-CD16 and PK-CD32 cells. (**C**) Enhanced ASFV replication in PK-CD16 and PK-CD32 cells upon treatment with anti-pA137R antibodies. The porcine anti-pA137R antibodies were diluted 50-fold, and 200 µL of the antibodies was combined with 200 µL of ASFV (multiplicity of infeciton = 1) at 37°C for 90 minutes to form a virus-antibodies complex. The PK-CD16, PK-CD32, or PK-15 cells were then infected with 100 µL of the virus-antibodies complex each. At 48 hpi, viral genome copies were quantified by qPCR. Statistical analysis was performed using one-way ANOVA followed by Student’s *t* test: ****, *P* < 0.0001; ***, *P* < 0.001; **, *P* < 0.01; *, *P* < 0.05. The data were presented as means and SDs.

We recently reported that the antibodies against Fc*γ*RII/CD32 and Fc*γ*RIII/CD16 can mitigate the ADE of ASFV infections in PAMs (17). To further clarify the roles of Fc*γ*RII and Fc*γ*RIII in enhancing ASFV replication, the PK-15 cell lines stably expressing CD16 or CD32 were established (Fig. 6A). Compared with PK-15 cells, the replication of ASFV was enhanced in PK-CD16 or PK-CD32 cells (Fig. 6B).

Subsequently, anti-pA137R antibodies were co-incubated with ASFV and used to infect PK-CD16 and PK-CD32 cells. The results demonstrated that the replication of ASFV in both PK-CD16 and PK-CD32 cells was significantly augmented after incubation with anti-pA137R antibodies (Fig. 6C), indicating that the anti-pA137R antibodies were implicated in driving the ADE of ASFV infection mediated by Fc*γ*RII and Fc*γ*RIII.

## DISCUSSION

ASF is a highly contagious disease caused by ASFV, posing a substantial threat to the global pig industry. Inoculation of pigs with certain candidate ASF vaccines has been observed to worsen clinical symptoms and significantly reduce survival time in challenged pigs (1–3). Moreover, antibodies against the ASFV structural protein A137R have been implicated in driving ADE of ASFV infection in PAMs. Given these findings, we hypothesized that the anti-pA137R antibodies may affect the pathogenesis of ASFV (17). In this study, these antibodies aggravated clinical signs, accelerated the progression to death, and increased virus titers in both blood and tissues. Specifically, the production of IFN-*α* was upregulated in the pigs with pre-existing anti-pA137R antibodies.

ASFV encodes an extensive proteome of over 165 proteins, encompassing 68 structural proteins (27). pA137R is a highly abundant viral protein that is expressed during the replication cycle of ASFV (28–30), and pA137R is a core component incorporated into the icosahedral capsid of ASFV particles (10). Anti-pA137R antibodies can bind to intracellular ASFV virions, which have infectivity similar to extracellular particles. Generally, the antibodies targeting the viral envelope or structural proteins are potentially able to drive ADE. For instance, a previous study has demonstrated that the antibodies against the E or prM protein can induce ADE of DENV infection (31). Notably, anti-pA137R antibodies enhance the replication of various ASFV genotypes in PAMs (17), thereby adding an intriguing layer of complexity to the understanding of ASFV pathogenesis. This study identified the ASFV pA137R to be associated with ADE in pigs.

A large number of previous studies have demonstrated that a variety of viruses, including DENV, ZIKV, and PRRSV, can cause the occurrence of ADE effects. DENV is a classical model of the ADE phenomenon. Research has found that in mouse models, antibodies produced after DENV infection can enhance viral replication through the ADE mechanism, leading to more severe pathological damage. Additionally, Fc*γ*Rs-mediated ADE significantly increases viral load and tissue pathological damage (32, 33). Furthermore, ADE significantly increased viral load and disease severity when rhesus monkeys were re-infected with different serotypes of DENV (34). ZIKV and DENV belong to the *Flaviviridae* family. Antigenic cross-reactivity between ZIKV and DENV has been found in both mice and rhesus monkeys to enhance ZIKV infection through the ADE mechanism, and this phenomenon is more obvious in human pregnant women (35–37). In immunodeficient mouse models, ADE significantly increased ZIKV replication and neurological damage (38). PRRSV is an important pathogen in pigs. Antibodies induced by PRRSV in pigs were found to enhance viral replication through the ADE mechanism, leading to more severe respiratory symptoms and reproductive disorders, and the Fc*γ*Rs-mediated ADE significantly increased viral load and lung pathological damage in pigs (39, 40). Baculovirus-expressed porcine epidemic diarrhea virus (PEDV) spike subunit vaccine (Bac-PEDV-S) can induce neutralizing antibodies in all immunized piglets. However, compared with the Bac-Ferritin control group, animals inoculated with the Bac-PEDV-S vaccine showed more severe clinical results after the challenge, significantly increasing the virus shedding from the jejunum contents. This indicates that the spike subunit vaccine enhances the pathogenicity of PEDV through an unclear antibody-dependent mechanism (41). These studies provide an important basis for the understanding of the ADE-mediated pathogenicity.

Non-NAbs induced by natural infection with a serotype of DENV may enhance the infection of another serotype of DENV, exacerbating the clinical severity of the disease (34). Studies have shown that pigs immunized with some ASF subunit vaccines and live virus-vectored vaccines show more severe clinical signs and die earlier than those of the non-immunized pigs after being challenged with virulent ASFV (2, 23). Additionally, our study found that after infection with the ASFV HLJ/18 strain, the presence of anti-pA137R antibodies accelerates the death of pigs. The ASFV infection elicits the production of anti-pA137R antibodies in pigs, which may increase the potential for ADE in pigs infected with ASFV in the field. Consequently, ADE may hold a significant role in the pathogenesis of ASFV. This study further confirmed the roles of the antibodies against pA137R in enhancing the pathogenicity of ASFV in pigs. In addition, it is needed to investigate whether antibodies against pA137R may potentially enhance the pathogenesis of genotype I and II recombinant ASFV strain.

Currently, no safe and effective ASF vaccine is commercially available globally, except for two conditionally approved vaccines in Vietnam. Analysis of different ASF vaccine development approaches reveals that inactivated vaccines provide hardly any protection, live-attenuated vaccines (such as ASFV-G-ΔI177L) have robust protection but have the risk of returning to strong virulence, subunit and viral vector vaccines demonstrate suboptimal efficacy, while DNA and mRNA vaccines remain in the laboratory research phase (42). Our analysis identifies that the challenge regarding vaccine safety lies in how to strike a balance between safety and immunogenicity. The fundamental question concerning efficacy revolves around how to scientifically design and deliver protective antigens. The proteins responsible for ADE may elicit non-NAbs, which significantly hinders vaccine and drug development (34). Therefore, the systematic identification of ADE-inducing antigens should remain a research priority. The involvement of ADE in ASFV infection presents a substantial challenge to the ASF vaccines. Given that the anti-pA137R antibodies are able to promote viral replication of ASFV *in vivo*, pA137R should be excluded in a safer and more effective ASF vaccine (12).

Furthermore, the viral infection in ADE circumstances modulates the host innate immune responses and alters the transcriptional levels of host molecules to facilitate viral replication (43). For example, the DENV infection, under the action of ADE, can promote the entry of the virus into target cells and inhibit the production of type I IFNs, thereby enhancing viral replication in the cells. Moreover, antibodies may potentiate the complement-activated inflammatory responses, thereby further exacerbating the severity of diseases caused by several viruses, such as respiratory syncytial virus and influenza A virus (44–46). It has been shown that both non-NAbs and highly diluted NAbs increase the replication of feline infectious peritonitis virus in macrophages and enhance the production of inflammatory cytokines, such as IL-1*β* and IL-6 and TNF-*α* (47). However, ADE of SARS-CoV-2 infection does not lead to aberrant cytokine production in macrophages (4). Additionally, highly lethal classical swine fever virus infection induces a high IFN-*α* response, which is associated with the depletion of lymphocytes (48). In contrast to the attenuated ASFV strains, the virulent ASFV infection induces abnormal IFNs in pigs undergoing acute ASF (49). Compared with the pigs infected with a virulent ASFV, a decrease in the level of IFN-*α* was observed in those infected with an ASFV mutant lacking the *MGF300-2R* gene, suggesting that IFN-*α* might play an important role in the pathogenesis of ASFV in pigs (50). The *A137R* gene deletion leads to the attenuation of the ASFV Georgia-2010 strain (13). In this study, our findings further indicate that the anti-pA137R antibodies upregulate the expression levels of cytokine IFN-*α* in pigs. Specifically, a correlation was observed between the IFN-*α* level and animal mortality. Therefore, as an ADE-related protein of ASFV, pA137R may also alter the host cell’s innate immune responses to contribute to cytokine production and pathogenicity, which will be investigated in the future.

The ADE of flaviviral infections is primarily attributed to the E and prM proteins. The subunit vaccine based on the ZIKV E-dimer lacking the prM protein is able to produce specific antibodies and provide full protection against ZIKV infection in mice (51). In addition, a nucleotide-modified DENV mRNA vaccine with the *prM* and *E* gene encapsulated in lipid nanoparticles (prM/E mRNA-LNP) induced NAbs and cellular immune responses in mice with reduced ADE (52). It has been reported that antibodies against SARS-CoV-2 nucleocapsid protein can produce a strong ADE effect, reduce virus clearance, and aggravate clinical symptoms (53, 54). It has been found that SARS-CoV-2 relies on the receptor-binding domain (RBD) of the S protein to bind to antibodies, and a subunit vaccine of the S protein lacking RBD can be designed to reduce ADE and prevent viral infection (54, 55). ADE associated with ASFV infection poses a safety concern for ASF vaccine development. Many vaccine strategies have been developed to eliminate ADE of virus infection. Future studies should prioritize the ASFV surface proteins involved in virus entry mechanisms to systematically assess their ADE potential through Fc*γ*R-dependent infection assays. For antigens exhibiting ADE activity, N-linked glycosylation is performed on non-critical linear epitopes, leveraging the glycan-mediated steric hindrance to block enhancing antibody binding while preserving conformational neutralizing epitopes. Concurrently, establishing a computational database of the ADE-associated epitopes from orthologous viruses would enable predictive identification of high-risk Fc*γ*R-binding motifs via the machine learning-assisted structural alignment. Subsequent rational antigen design should excise or mutate these Fc*γ*R-engaging domains to develop ADE-proof subunit vaccines. Alternatively, T cell-based vaccine platforms could circumvent humoral ADE risks by focusing on conserved MHC-I/II-restricted epitopes with minimal B cell immunogenicity. Additionally, it is necessary to identify other ADE-associated ASFV proteins for providing clues to the development of safer vaccines.

In conclusion, antibodies against pA137R produced in pigs can enhance the pathogenicity of ASFV in pigs, providing novel insights into the role of anti-ASFV antibodies and informing the rational design of novel ASF vaccines.

## MATERIALS AND METHODS

### Cells and viruses

PAMs and WSL cells were cultured in RPMI-1640 medium (catalog no. C11875500BT, Gibco) supplemented with 10% fetal bovine serum (catalog no. 10091148, Gibco) and 2% penicillin-streptomycin (catalog no. 15140122, Gibco). All cells were cultured in a 37°C incubator with 5% CO_2_. The genotype II highly virulent ASFV HLJ/18 strain (GenBank no. MK333180.1) was propagated as described previously (56–58).

### qPCR

To evaluate viral loads, the ASFV genome was extracted from samples using the MagaBio plus viral DNA purification kit (catalog no. 9109, BioFlux) following the manufacturer’s protocols. The ASFV genome was quantified on a QuantStudio system (Applied Biosystems, USA) as described previously.

### IFA

WSL cells in 24-well cell culture plates were infected with the ASFV HLJ/18 strain and subsequently incubated in a 5% CO_2_-supplemented incubator at 37°C for 48 hours (59). Following incubation, the cells were fixed using 4% paraformaldehyde and permeabilized with 0.2% Triton X-100 (catalog no. 9036–19-5, Sigma-Aldrich). Subsequently, the cells were incubated with either porcine anti-pA137R antibodies or negative sera diluted at a ratio of 1:100 at 37°C for 2 hours, followed by four thorough washes with PBS containing 0.05% Tween-20 (PBST). Thereafter, the cells were incubated with fluorescein isothiocyanate-conjugated goat anti-pig IgG (catalog no. 114–095-003, Jackson), which was diluted at a ratio of 1:300 for 1 hour. Finally, all samples underwent four additional washes with PBST and were examined under an inverted fluorescence microscope (EVOS FL, USA).

### Indirect ELISA

Recombinant pA137R was diluted with PBS to 6.25 µg/mL, added to the ELISA microplates, and incubated overnight at 4°C. The microplates underwent washing four times with PBST. Subsequently, the plates were blocked with 200 µL of blocking buffer (Surmodics, USA) at 37°C for 1 hour. Afterward, the ASFV-convalescent sera were diluted in PBS at a ratio of 1:500 and incubated on the plates at 37°C for 1 hour. After five washes with PBST, horseradish peroxidase-conjugated goat anti-pig IgG (catalog no. F1638-2ML, Sigma-Aldrich), diluted to 1:10,000 in the StabilZyme SELECT Stabilizer (catalog no. SZ03-1000, Surmodics), was added to each well and incubated at 37°C for 1 hour. Subsequent to five additional washes with PBST, 100 µL of the chromogenic substrate solution (TMB, catalog no. 34022, Thermo Fisher) was added to the plates and incubated for 15 minutes. The enzymatic reaction was terminated by adding 50 µL of 2 M H_2_SO_4_. Finally, the optical density (OD) values at 450 nm (OD_450nm_) of the reactions were measured using a microplate reader (Biotek, USA).

### Animal experiments

Six 7-week-old SPF piglets were randomly assigned to two groups for intramuscular injection: three piglets inoculated with the recombinant pA137R protein, and three piglets inoculated with PBS. The piglets were intramuscularly challenged with 10^3.0^ HAD_50_ of the ASFV HLJ/18 strain. All pigs were monitored daily for rectal temperature, survival, and clinical signs. The piglets were monitored daily for clinical signs before feeding, including anorexia, lethargy, fever, and emaciation. Serum samples and different swabs were collected at 0, 3, 7, and 10 dpc to quantify viral loads. The viral loads in the organs of each necrotic pig, including the heart, liver, spleen, lungs, kidneys, tonsils, and six lymph nodes, were quantified by qPCR.

### Cytokine assay

The cytokines in the plasma from the experimental piglets were quantified by porcine IFN-*α* (catalog no. ml002376, MLBio), IFN-*β* (catalog no. ml002398, MLBio), TNF-*α* (catalog no. ml002360, MLBio), and IL-1*β* (catalog no. ml0025973, MLBio) ELISA kit according to the manufacturer’s instructions.

### Establishment of PK-15 cell lines stably expressing CD16 or CD32

The recombinant lentivirus was generated by transfecting a mixture of 10.5 μg of either pLVX-EGFP-CD16 or pLVX-EGFP-CD32, with 7 μg of pSPAX2 and 3.5 μg of pMD2.G plasmids, into HEK293T cells using X-treme GENE HP DNA transfection reagent (catalog no. 6366236001, Sigma-Aldrich). Following transfection, the lentivirus was harvested after a 48-hour incubation. Subsequently, the lentivirus was used to infect PK-15 cells, resulting in the PK-CD16 and PK-CD32 cell lines. The EGFP-expressing cells were isolated by flow cytometry (Sony, Japan) and subcultured for 10 generations. Subsequently, these cells that were overexpressing CD16 or CD32 were used for further experiments.

### Statistical analysis

The SPSS 22.0 software (SPSS Software, Inc.) was used to analyze all data. The statistical significance of differences between groups was assessed using Student’s *t* test. An unadjusted *P*-value of less than 0.05 was considered to be significant.

## Data Availability

All the data presented in this study are available.
